# The Transmission of Tuberculosis in Childhood

**Published:** 1915-12

**Authors:** 


**The Transmission of Tuberculosis in Childhood.** W. H. Park, M. D., Arch. of Ped., July. Combined tabulation of cases reported and own series of cases.*

| Diagnosis | Adults 16 Years and Over | | Children 5 to 16 Years | | Children Under 5 Years | |
|---|---|---|---|---|---|---|
| | Human | Bovine | Human | Bovine | Human | Bovine |
| Pulmonary tuberculosis | 778 | 3 | 14 | — | 35 | 1 |
| Tuberculous adenitis, axillary or inguinal | 3 | — | 4 | — | 2 | — |
| Tuberculous adenitis, cervical | 36 | 1 | 36 | 22 | 15 | 24 |
| Abdominal tuberculosis | 16 | 4 | 8 | 9 | 10 | 14 |
| Generalized tuberculosis, alimentary origin | 6 | 1 | 3 | 4 | 17 | 15 |
| Generalized tuberculosis | 29 | — | 5 | 1 | 74 | 7 |
| Generalized tuberculosis, including meninges, alimentary origin | — | — | 1 | — | 5 | 10 |
| Generalized tuberculosis, including meninges | 5 | — | 10 | — | 76 | 1 |
| Tubercular meningitis | 1 | — | 3 | — | 28 | 4 |
| Tuberculosis of bones and joints | 32 | 1 | 41 | 3 | 27 | — |
| Genito-urinary tuberculosis | 22 | 1 | 2 | — | — | — |
| Tuberculosis of skin | 10 | 3 | 4 | 6 | 2 | — |
| Miscellaneous cases: | | | | | | |
| Tuberculosis of tonsils | — | — | — | 1 | — | — |
| Tuberculosis of mouth and cervical nodes | — | 1 | — | — | — | — |
| Tuberculous sinus or abscess | 2 | — | — | — | — | — |
| Sepsis, latent bacilli | — | — | — | — | 1 | — |
| Totals | 940 | 15 | 131 | 46 | 292 | 76 |

Mixed or double infections, 11 cases.

TOTAL CASES—1,511, of which 478 were cases tested by us.

*Journal of Medical Research, Vol. XXVII., No. 1.

